# Machine Learning for Predicting Zearalenone Contamination Levels in Pet Food

**DOI:** 10.3390/toxins16120553

**Published:** 2024-12-23

**Authors:** Zhenlong Wang, Wei An, Jiaxue Wang, Hui Tao, Xiumin Wang, Bing Han, Jinquan Wang

**Affiliations:** 1 Key Laboratory of Feed Biotechnology, Ministry of Agriculture and Rural Affairs, Institute of Feed Research, Chinese Academy of Agricultural Sciences, No. 12 Zhongguancun South Street, Beijing 100081, China; wangzhenlong02@caas.cn (Z.W.);; 2 Laboratory of Pet Nutrition and Food, Institute of Feed Research, Chinese Academy of Agricultural Sciences, No. 12 Zhongguancun South Street, Beijing 100081, China

**Keywords:** mycotoxin, zearalenone, machine learning, E-nose, pet food

## Abstract

Zearalenone (ZEN) has been detected in both pet food ingredients and final products, causing acute toxicity and chronic health problems in pets. Therefore, the early detection of mycotoxin contamination in pet food is crucial for ensuring the safety and well-being of animals. This study aims to develop a rapid and cost-effective method using an electronic nose (E-nose) and machine learning algorithms to predict whether ZEN levels in pet food exceed the regulatory limits (250 µg/kg), as set by Chinese pet food legislation. A total of 142 pet food samples from various brands, collected between 2021 and 2023, were analyzed for ZEN contamination via liquid chromatography–tandem mass spectrometry. Additionally, the “AIR PEN 3” E-nose, equipped with 10 metal oxide sensors, was employed to identify volatile compounds in the pet food samples, categorized into 10 different groups. Machine learning algorithms, including liner regression, k-nearest neighbors, support vector machines, random forests, XGBoost, and multi-layer perceptron (MLP), were used to classify the samples based on their volatile profiles. The MLP algorithm showed the highest discrimination accuracy at 86.6% in differentiating between pet food samples above and below the ZEN threshold. Other algorithms showed moderate accuracy, ranging from 77.1% to 84.8%. The ensemble model, which combined the predictions from all classifiers, further improved the classification performance, achieving the highest accuracy at 90.1%. These results suggest that the combination of E-nose technology and machine learning provides a rapid, cost-effective approach for screening ZEN contamination in pet food at the market entry stage.

## 1. Introduction

Companion animals play an important role in people’s lives, with extensive research highlighting the positive effects of pet ownership on psychological well-being and physical health [1]. For example, interacting with pets is associated with reduced cardiovascular disease risk [2] and improved emotional development in children by fostering responsibility and self-confidence [3]. As societal attitudes evolve and pets become integral family members, ensuring their health and safety has become a priority for pet owners. This, in turn, has driven rapid growth in the pet food industry while intensifying concerns over pet food safety, particularly regarding mycotoxin contamination [4].

Mycotoxins are toxic secondary metabolites synthesized by various fungi [5], which can contaminate pet food and pose significant health risks to pets, leading to serious emotional and financial concerns for pet owners [6]. Main mycotoxins, such as aflatoxins (AFs), zearalenone (ZEN), deoxynivalenol (DON), and fumonisin B1 (FB1), have been detected in pet food ingredients and finished products [7,8], causing acute poisoning and chronic health issues, including immune suppression, liver damage, kidney toxicity, and cancer [9]. Recent studies underscore the prevalence of mycotoxin contamination in commercial pet food. For example, a survey of 55 dog food and 34 cat food samples revealed that 99% contained mycotoxins, with up to 16 different mycotoxins detected per sample [10]. Similarly, another study in China revealed that 96.9% of commercial dry pet foods were contaminated with at least three different mycotoxins [11].

Among these, ZEN, produced by *Fusarium* species, is particularly concerning due to its prevalence in grain-based ingredients commonly used in pet foods [12]. Given that pet food often contains multiple grains, ZEN contamination is almost unavoidable [13]. Long-term exposure to ZEN can harm pets’ reproductive systems and lead to reduced body weight gain in female dogs [14]. Therefore, it is essential to monitor ZEN contamination in pet food before it reaches the market to ensure the health and safety of companion animals. Traditional detection methods for ZEN, such as chromatographic techniques (e.g., HPLC and LC-MS) and immunoassays (e.g., ELISA), are widely utilized due to their high sensitivity and specificity [15,16]. However, these methods often require complex sample preparation and expensive instrumentation and are time-consuming, limiting their practicality for routine and rapid screening in industrial settings.

To address these limitations, recent studies have explored alternative detection techniques, including electronic nose (E-nose) systems that analyze the volatile compounds associated with mycotoxin contamination. By integrating machine learning algorithms with E-nose technology, it is possible to construct predictive models capable of rapid, non-invasive detection of mycotoxins [17,18]. In this study, we aim to develop machine learning-based predictive models using E-nose odor data to accurately classify ZEN contamination levels in pet food samples. This novel approach will provide an efficient and innovative alternative to traditional detection methods.

## 2. Results

### 2.1. ZEN Analysis in Pet Food

A total of 142 pet food samples from various brands in China, collected between 2021 and 2023, were analyzed to determine ZEN concentrations using ultra-performance liquid chromatography coupled with tandem mass spectrometry, and the descriptive statistics are presented in Table 1. ZEN concentrations ranged from undetectable levels to a maximum of 4133 μg/kg, with a median concentration of 177.5 μg/kg and a mean of 392.1 μg/kg (SD = 626.8 μg/kg). Notably, 54 samples (38.0%) exceeded the legal threshold of 250 μg/kg (specified by Chinese pet food legislation) for ZEN contamination, while the remaining 88 samples (62.0%) had concentrations below or equal to this limit. These findings indicate a significant level of ZEN contamination in the tested pet food products, with over one-third of the samples surpassing the regulatory limit, posing a potential health risk to companion animals due to ZEN exposure.

### 2.2. E-Nose Data Analysis

Pet food can emit a variety of volatile organic compounds with odors such as aromatics, ammonia, and sulfur compounds. In our study, volatile compounds in pet food samples were analyzed using the E-nose, and the results are presented in Table 2. A total of 10 volatile compounds were detected in the samples, with R7 (reacts with sulfur compounds) being the most abundant, followed by R9 (aromatic compounds) and R6 (sensitive to methane). The samples were categorized based on ZEN contamination levels, being assigned a label of 1 if ZEN concentrations exceeded 250 μg/kg and a label of 0 if ZEN concentrations were at or below this threshold.

**Figure 1:**
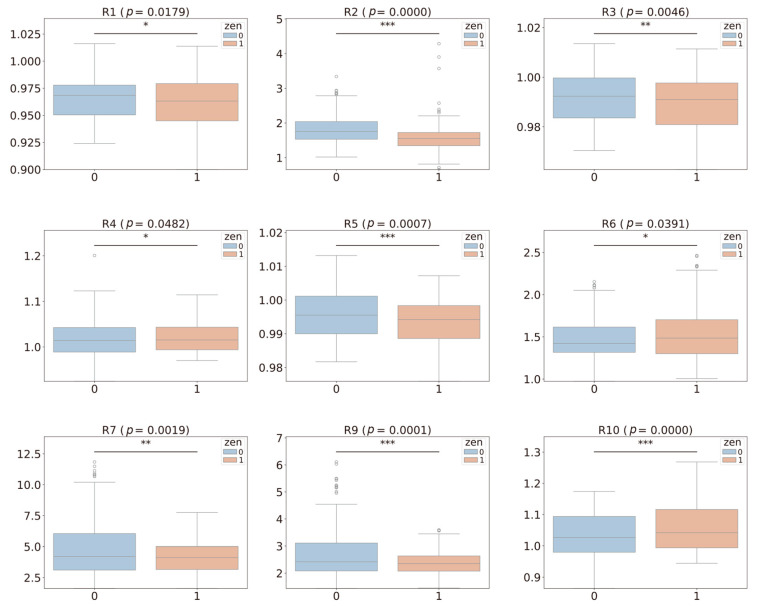
Box plots and statistical analysis of ZEN contamination levels (1: >250 μg/kg, 0: ≤250 μg/kg) and different volatile compounds. The asterisks indicate a significant difference between the two groups (* *p* < 0.05, ** *p* < 0.01, *** *p* < 0.001). The circles represent observations outside the upper or lower error bars of the box plot.

As shown in Figure 1, box plots were generated to illustrate the relationship between each volatile compound and ZEN contamination grouping. We performed a *t*-test to analyze the differences in volatile compounds between the contaminated and uncontaminated samples. The results indicated significant differences (*p* < 0.05) for all volatile compounds, except for R8 (detects alcohols), between the ZEN-exceeding and non-exceeding groups. These findings suggest that the volatile compound data captured by the E-nose could serve as valuable indicators for discriminating between pet food samples with ZEN contamination above and below the legal threshold.

### 2.3. Classification Model

Six machine learning algorithms, including logistic regression (LR), k-nearest neighbors (k-NN), support vector machine (SVM), random forest (RF), extreme gradient boosting (XGBoost), and multi-layer perceptron (MLP), were used to classify pet food samples based on ZEN contamination levels as specified by Chinese pet food legislation (250 µg/kg). As shown in Table 3, each model performed well using volatile compound data, with classification accuracies ranging from 77.1% to 86.6%. Among these, the MLP classifier showed the highest individual accuracy at 86.6%, with an F1 score of 79.8%, recall of 83.8%, and precision of 77.9%. The ensemble model, which combined predictions from all classifiers, further enhanced the classification performance, achieving the highest accuracy at 90.1% and a precision of 92.3%. These results indicate that while individual models such as MLP and RF performed well, the ensemble model provided the most robust classification for detecting ZEN contamination in pet food samples.

### 2.4. Feature Importance

**Figure 2:**
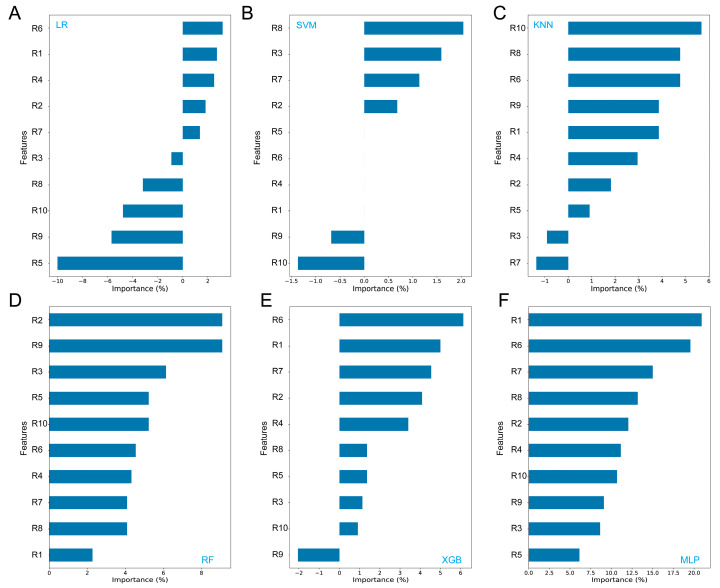
Feature importance analysis of six machine learning models. (**A**) logistic regression (LR); (**B**) support vector machine (SVM); (**C**) k-nearest neighbors (k-NN); (**D**) random forest (RF); (**E**) extreme gradient boosting (XGB); (**F**) multi-layer perceptron (MLP).

Based on the feature importance analysis, we observed notable differences in the ranking of the input features’ importance across different models. For the LR, XGB, and MLP models, the features R6 (sensitive to methane) and R1 (sensitive to aromatic compounds) were identified as the most important features (Figure 2A,E,F). We also observed that approximately half of the features (such as R5, R9, and R10) are largely unrelated to the LR and SVM models, showing negative importance values. This could explain why these models exhibit lower accuracies compared to others (Table 3). For the SVM and KNN models, R8 (sensitive to alcohols) and R10 (sensitive to long-chain alkanes) were found to be the most important features, respectively (Figure 2B,C). R8 was also the primary feature of KNN. For the RF model, we see that features such as R2 (sensitive to nitrogen oxides and ozone) and R9 (sensitive to aromatic compounds and sulfur organic compounds) ranked highly. These findings demonstrate the variability in feature ranking, which highlights the necessity of using appropriate models for accurate ZEN contamination classification.

### 2.5. Model Validation

**Figure 3:**
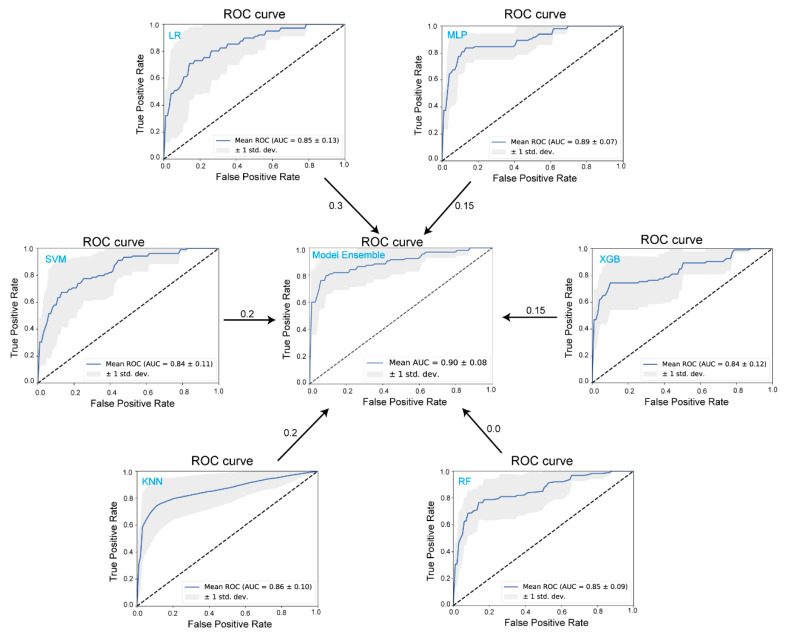
ROC curves of six machine learning models: logistic regression (LR), support vector machine (SVM), k-nearest neighbors (k-NN), random forest (RF), extreme gradient boosting (XGB), multi-layer perceptron (MLP), and ensemble models for classifying ZEN contamination in pet food samples. The dashed line represents the random classification baseline. ROC curves above the baseline indicate that the classifiers possess discriminatory ability superior to random guessing. The shaded area illustrates the variation in model performance across five independent experiments. The numbers shown by the arrows represent the contribution weights of each individual model to the ensemble model.

To evaluate the performance of these models, we calculated the cross-validation ROC curves and their corresponding AUC values. The ROC curves in Figure 3 highlight the performance of six individual models—LR, k-NN, SVM, RF, XGB, and MLP—along with the ensemble model. Although the LR model shows a relatively lower accuracy (77.1%), its AUC is not the lowest among the models. In fact, it contributes a weight of 0.3 to the ensemble, suggesting that the LR model provides crucial information for distinguishing between ZEN-contaminated and uncontaminated samples. In contrast, the MLP model, which achieved the best individual performance with an accuracy of 86.6% and an AUC of 0.89, contributes only 0.15 to the ensemble. Other models, such as SVM, k-NN, and XGBoost, contribute weights between 0.15 and 0.2, reflecting their moderate influence. Despite RF’s moderate individual performance (accuracy = 84.8%, AUC = 0.85 ± 0.09), it contributed no weight to the ensemble model. This suggests that RF’s decision paths may be redundant with other models, such as XGBoost or k-NN, or less beneficial for improving the ensemble’s overall discrimination power. Ultimately, as shown in the ensemble’s ROC curve (AUC = 0.90 ± 0.08), combining these models leads to the best overall performance, with the highest accuracy at 90.1% and a precision of 92.3%.

**Figure 4:**
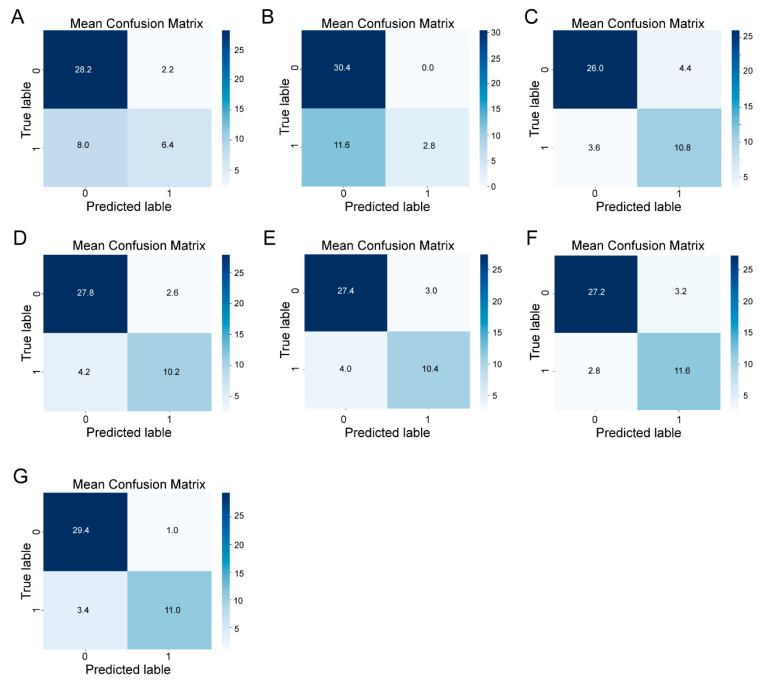
Confusion matrices for different machine learning models applied to the classification of contamination in pet food samples. The models include the following: (**A**) logistic regression (LR), (**B**) support vector machine (SVM), (**C**) k-nearest neighbors (k-NN), (**D**) random forest (RF), (**E**) extreme gradient boosting (XGB), (**F**) multi-layer perceptron (MLP), and (**G**) ensemble models. The rows correspond to the actual class labels (0 for uncontaminated, 1 for contaminated), and the columns represent the predicted labels. The numbers in each cell indicate the proportion of samples for each prediction outcome. Correct classifications are in the upper-left quadrants (true negatives) and lower-right quadrants (true positives). Misclassifications are in the upper-right quadrants (false positives) and lower-left quadrants (false negatives).

In the confusion matrices (Figure 4), we use “1” and “0” to represent positive samples (ZEN level > 250 μg/kg) and negative samples (ZEN level ≤ 250 μg/kg). For each matrix, the rows represent the actual class of the samples (true labels), while the columns correspond to the predicted class (predicted labels). LR (Figure 4A) and SVM (Figure 4B) show a tendency to correctly predict negative samples but are less effective with positive samples, indicating lower recall for the contaminated class (Table 1). KNN (Figure 4C) has moderate performance, with higher misclassifications in both positive and negative classes compared to the other models. RF (Figure 4D), XGB (Figure 4E), and MLP (Figure 4F) deliver better performance with relatively high precision and recall across both classes. The ensemble model (Figure 4G) shows the highest classification accuracy among all the models. The ensemble model correctly predicted 96.7% of the negative samples (true negatives) and 76.4% of the positive samples (true positives), indicating balanced and robust classification performance. In summary, while individual models provide varying levels of accuracy, the ensemble model offers the most balanced and accurate classification, benefiting from the combined predictions of multiple algorithms.

## 3. Discussion

The detection of mycotoxins in pet food is vital to safeguarding the health and well-being of pets, as these toxins can lead to serious health issues, such as immune suppression, organ damage, and even cancer [9]. Over the years, many analytical techniques have been developed to detect mycotoxins. Recent advancements in techniques used to analyze and quantify major mycotoxins are summarized in a review [19]. At the pet food industry level, continuous on-site monitoring of product quality and safety is imperative. Therefore, adopting rapid, cost-effective, high-throughput screening methods for mycotoxin detection is crucial for making timely decisions regarding the acceptance or rejection of product batches [20,21]. The E-nose is a valuable tool for monitoring quality and safety throughout the feed production chain [22]. It can predict mycotoxin contamination by detecting alterations in the volatile substances emitted by pet food. Particularly, the combination of electronic nose technology with machine learning algorithms has been successfully applied to distinguish AFB1, DON, and FB contamination in maize and wheat [17,18].

Our study used a similar approach to assess ZEN contamination in pet food. In total, 142 pet food samples were analyzed using the E-nose. We analyzed the presence of major mycotoxins such as DON, AFB1, ZEN, and FBs in these samples. However, due to a data imbalance between contaminated and uncontaminated samples for DON, AFB1, and FB1, these data were not suitable for model training [23]. In contrast, the relatively balanced distribution of ZEN-contaminated and uncontaminated samples (54 contaminated:88 uncontaminated, 1:1.63 ratio) provided a strong foundation for reliable machine learning-based prediction. Additionally, we analyzed 10 different volatile compound profiles (odor data) to determine their relevance to ZEN contamination in pet food samples. Of these, nine odor profiles exhibited significant differences between the ZEN-contaminated (label = 1) and uncontaminated (label = 0) samples, indicating their strong correlation with ZEN levels, providing a solid basis for the binary classification of ZEN contamination through machine learning models. Although R8’s profile (detects alcohols and partially aromatic compounds) did not show statistically significant differences (*p* > 0.05) between the two groups, we still included it as an input feature in the machine learning models. This decision was based on the potential for machine learning algorithms to capture hidden patterns or interactions across all odor types, including those that may seem less relevant in isolation [24].

In our study, six machine learning models were employed for classifying ZEN contamination in pet food samples, with the overall accuracy ranging from 77.1% to 86.6%. Among these, the MLP model demonstrated the highest individual accuracy at 86.6%, indicating its superior ability to other models. This result is consistent with findings from Leggieri et al., who applied MLP, LR, and discriminant analysis to analyze AFB1 and FB contamination in maize, with MLP outperforming the other models, achieving accuracy rates of 78% for AFB1 and 77% for FBs [18]. The superior performance of the MLP model can be attributed to its ability to capture and model complex, non-linear relationships within the volatile compound data from pet food samples [25].

Rather than relying on any single model from previous reports [17,18], we introduced an ensemble model to predict ZEN contamination based on the E-nose data. The ensemble model achieved the highest overall performance with an accuracy of 90.1%, significantly surpassing all standalone models. The superior performance of the ensemble model can be attributed to its ability to combine the complementary strengths of multiple classifiers, thereby improving accuracy and generalization [26]. Although the LR model shows a relatively lower accuracy (77.1%), it surprisingly contributed the most weight (0.3) to the ensemble model. This suggests that, despite LR’s weaker individual performance, its linearity may provide critical baseline insights that help the ensemble model to better distinguish between contaminated and uncontaminated samples. In contrast, the MLP model, which achieves the best individual performance with an accuracy of 86.6%, contributes only 0.15 to the ensemble model. While MLP effectively captures more complex, non-linear interactions between features, the ensemble may rely less on it because its complexity, when combined with simpler models like LR, is sufficient to enhance performance without over-complicating the prediction process.

According to Chinese pet food regulations (Ministry of Agriculture and Rural Affairs of China, Announcement No. 20, 2020), permissible levels of ZEN in pet food are set at 250 µg/kg for adult dogs and cats and 150 µg/kg for juvenile dogs and cats. In this study, we applied the regulatory threshold of 250 µg/kg for dogs and cats. Although the threshold of 250 µg/kg complies with the general pet food standards, it does not effectively predict risks for juvenile dogs and cats posed by samples with ZEN levels between 150 and 250 µg/kg. This limitation arises mainly from our preliminary experiments, where using 150 µg/kg as the cut-off value resulted in models with relatively low accuracy (<70%). Future improvements in accuracy will require the accumulation of more samples and the fine-tuning of model parameters. Additionally, another certain limitation of our study should be acknowledged. The sample size was relatively small (142 samples), and expanding the dataset would be crucial for enhancing the generalizability of the models. Unlike previous approaches that increased data size through oversampling or feature engineering [27], in this study we addressed the issue by conducting three repeated measurements for each sample. To prevent data leakage, we employed a “group shuffle split” method, ensuring that all three measurements of each sample were assigned either to the training or the test set. This approach not only mitigated the risk of overfitting but also allowed us to effectively increase the amount of trainable data. Moreover, while the MLP and ensemble models performed well, further refinement of model parameters through additional hyperparameter tuning and feature engineering could potentially enhance the classification accuracy. In conclusion, the integration of E-nose technology with machine learning offers a rapid, efficient, and cost-effective approach for ZEN contamination screening in pet foods. This method holds great promise for industrial application, providing a means for timely decision-making regarding the safety of pet food products before they reach the market.

## 4. Conclusions

This study demonstrates the effectiveness of combining E-nose technology with machine learning algorithms for the rapid detection of ZEN contamination in pet food. Among the models tested, the MLP exhibited the highest individual accuracy (86.6%), while the ensemble model achieved the best overall performance, with an accuracy of 90.1%. These results highlight the potential of this approach as a fast, cost-effective screening tool for identifying mycotoxin contamination, offering significant benefits for ensuring pet food safety at the market entry stage.

## 5. Methods

### 5.1. Sample Preparation and E-Nose Analysis

A total of 142 pet food samples were collected from various brands in China between 2021 and 2023. These samples were ground into powder, and three subsamples (10 g each) from each sample were placed into 50 mL round-bottom flasks to equilibrate the volatile compounds in the headspace for 12 h at 25 °C prior to analysis. A portable AIRPEN3 E-nose, equipped with an array of 10 different metal oxide sensors, was then used to analyze the volatile compounds as described previously [18]. Initially, charcoal-filtered environmental air was introduced into the sensor array at a flow rate of 400 mL/min until the response curves for each sensor stabilized (approximately 60 s). Signals from each sensor were recorded at a frequency of 1 Hz, and the E-nose output signals were calculated as the ratio G/G0, where G represents the electronic conductivity of each sensor when detecting the sampled gas, and G0 represents the electronic conductivity in clean air. After recording the sample gas signals, charcoal-filtered air was again pumped into the gas path and chamber at a flow rate of 400 mL/min for 60 s to clean the system and return the sensor signals to the baseline. The E-nose sensor outputs were used in a subsequent data analysis, which was aimed at assessing potential associations with ZEN contamination.

### 5.2. ZEN Analysis

For ZEN content analysis in pet food, a 25 g portion of each pet food sample was extracted with 100 mL of 50% acetonitrile solution using a shaker set at 250 rpm for 1 h. The extract was then filtered, and 100 µL of acetic acid was added to 2 mL of the filtered supernatant. Subsequently, 750 μL of this mixture was purified using a MycoSpin™ 400 multifunctional purification column (Romer Labs, Getzersdorf, Austria). The analytes were quantified by ultra-performance liquid chromatography–tandem mass spectrometry (UPLC-MS/MS) following derivatization [28]. The limit of detection (LOD) was 3 µg/kg, and the limit of quantification (LOQ) was 10 µg/kg.

Chromatographic separation was performed on a Phenomenex Gemini C18 column (4.6 × 150 mm, 5 μm) at 40 °C with a flow rate of 1 mL/min and an injection volume of 20 µL. Mobile phase A used 2 mmol/L ammonium acetate with 0.5% acetic acid in water, and phase B used 2 mmol/L ammonium acetate with 0.5% acetic acid in methanol. The elution conditions were as follows: 90% A and 10% B from 0 to 1 min; 3% A and 97% B from 14 to 15 min; and 90% A and 10% B from 15.1 to 20 min. Mass spectrometry detection was performed using electrospray ionization in both positive- and negative-ion modes. The ion source temperature was set at 650 °C for positive-ion mode and 600 °C for negative-ion mode. Curtain gas pressure was maintained at 35 psi, with a medium collision gas level. The electrospray voltage was set at 5000 V in positive-ion mode and −4500 V in negative-ion mode, while the Gas1 and Gas2 pressures were 60 psi and 65 psi, respectively.

### 5.3. Data Processing

ZEN concentrations were used to classify the samples into “contaminated” and “uncontaminated” groups. A threshold of 250 µg/kg was set, resulting in two distinct clusters. Samples with ZEN concentrations exceeding the threshold were labeled “1”, while those with concentrations at or below the threshold were labeled “0”. Odor data, consisting of ten variables, were utilized as input for the machine learning models. To ensure uniform scaling of all features, the values of each continuous variable were standardized using the “MinMaxScaler” function from the Scikit-Learn (1.6.0) library. Each pet food sample was independently analyzed three times with the E-nose, with each measurement corresponding to the same class label (1 or 0). Then, “group shuffle split” was applied to ensure no E-nose measurement from the same pet food sample appeared in both the training and validation sets. To ensure rigorous model evaluation and reduce bias in our results, we employed a randomized splitting strategy. Specifically, we randomly split the dataset into 10 parts 5 times to ensure variability across the splits. For each time, we took 90% of the data as the training set and reserved the remaining 10% as the test set (n_splits = 5, test_size = 0.1).

### 5.4. Model Construction and Evaluation

To develop and evaluate predictive models for classifying pet food samples based on volatile compound data and their association with ZEN contamination, we implemented six machine learning algorithms: linear regression (LR), support vector machine (SVM), k-nearest neighbors (k-NN), random forest (RF), extreme gradient boosting (XGB), and multi-layer perceptron (MLP). These models were selected due to their robustness and efficacy in handling classification tasks involving complex and multivariate data [29].

Specifically, the LR was chosen for its simplicity and efficiency in binary classification, offering interpretable results through the estimation of class probabilities based on linear relationships between features. The SVM was included due to its strong performance in high-dimensional spaces and ability to create hyperplanes that maximize the margin between different classes. The KNN algorithm was chosen for its simplicity and effectiveness in handling small datasets, relying on the principle of proximity-based classification. The RF model, an ensemble learning method, was selected for its capability to handle high-dimensional data and reduce overfitting by aggregating multiple decision trees. XGB was employed as an efficient and scalable machine learning system, known for its ability to handle classification tasks with high accuracy by utilizing gradient boosting frameworks. Finally, the MLP, a type of artificial neural network, was used for its capacity to model complex patterns and interactions between features. Additionally, an ensemble model combining the predictions of multiple algorithms was also implemented to further enhance classification accuracy by leveraging the strengths of different models. The ensemble model aimed to improve overall performance by reducing the variance and bias inherent in individual models.

All models were implemented within the Scikit-Learn framework using Python in a Jupyter Notebook environment. Hyperparameter tuning for each model was conducted using cross-validation to optimize performance, ensuring the best balance between bias and variance. Feature importance was analyzed using permutation importance to identify and rank the most influential features. The performance of the models was evaluated based on standard classification metrics, including accuracy, precision, recall, and F1 score:

$$Accuracy=\frac{TP+TN}{TP+TN+FP=FN}$$

$$Precision=\frac{TP}{TP+FP}$$

$$Recall=\frac{TP}{TP+FN}$$

$$F1-Score=2\times \frac{Precision\times Recall}{Precision+Recall}$$

where TP = true positive; TN = true negative; FP = false positive; and FN = false negative. An area under the receiver operating characteristic curve (AUC-ROC) was used to provide a comprehensive assessment of their predictive abilities in classifying ZEN-contaminated pet food samples.

## Figures and Tables

**Table 1:** ZEN content in pet food samples analyzed by UPLC-MS/MS.

No. of Samples	No. of Samples > 250 μg/kg	No. of Samples ≤ 250 μg/kg	Min (μg/kg)	Max (μg/kg)	Median (μg/kg)	Mean (μg/kg)	SD (μg/kg)
142	54	88	0	4133	177.5	392.1	626.8

**Table 2:** Analysis of 10 volatile compounds in pet food.

	R1	R2	R3	R4	R5	R6	R7	R8	R9	R10	ZEN
Count	142	142	142	142	142	142	142	142	142	142	142
Mean	0.963	1.746	0.991	1.016	0.995	1.517	4.530	1.384	2.576	1.040	392.1
Std	0.020	0.424	0.010	0.034	0.007	0.279	1.904	0.161	0.761	0.076	626.8
Min	0.906	0.730	0.965	0.935	0.977	1.069	1.812	1.109	1.559	0.871	0.000
50%	0.967	1.674	0.992	1.016	0.995	1.455	4.180	1.339	2.401	1.037	177.5
Max	1.005	3.921	1.010	1.101	1.009	2.362	10.917	1.841	5.526	1.245	4133.0

R1: Detects aromatic compounds. R2: Sensitive to negative signals, such as nitrogen oxides and ozone. R3: Primarily detects ammonia; also responds to aromatic compounds. R4: Mainly detects hydrogen, selectively for respiratory gases. R5: Responds to alkanes, aromatic compounds, and less polar compounds. R6: Sensitive to environmental methane (around 10 ppm), with a broad detection range, similar to R8. R7: Reacts to sulfur compounds (H2S at 0.1 ppm), also sensitive to terpenes and sulfur organic compounds like limonene and pyrazine. R8: Detects alcohols and some aromatic compounds; has a broad range. R9: Sensitive to aromatic compounds and sulfur organic compounds. R10: Reacts with high concentrations (>100 ppm), sometimes especially methane.

**Table 3:** Performance measurements of each classification model.

Models	F1 Score	Recall	Precision	Accuracy
LR	54.6%	47.6%	72.5%	77.1%
SVM	30.7%	19.5%	80.0%	74.0%
KNN	72.5%	78.6%	70.2%	82.1%
RF	75.6%	74.0%	79.2%	84.8%
XGB	74.5%	74.4%	75.9%	84.3%
MLP	79.8%	83.8%	77.9%	86.6%
Model Ensemble	84.6%	79.7%	92.3%	90.1%

## Data Availability

The data presented in this study are only available upon request from the corresponding author due to policy restrictions. Some of the data are sourced from the China pet food risk early warning system, which requires formal approval for access.
